# Weight gain secondary to the use of oral Janus kinase inhibitors: A systematic review and meta-analysis

**DOI:** 10.1016/j.jdin.2024.11.009

**Published:** 2024-12-19

**Authors:** Grace Xiong, Eric Yu, Martin Heung, Jaehyeong Yang, Megan Lowe, Mohannad Abu-Hilal

**Affiliations:** a Michael G. DeGroote School of Medicine, McMaster University, Hamilton, Canada; b Faculty of Health Sciences, McMaster University, Hamilton, Canada; c Queen's University School of Medicine, Queens University, Kingston, Canada; d Division of Dermatology, McMaster University, Hamilton, Canada

**Keywords:** abrocitinib, baricitinib, Janus kinase inhibitors, medical dermatology, ruxolitinib, tofacitinib, upadacitinib, weight changes

## Abstract

Oral Janus kinase inhibitors (JAKi) are increasingly used in dermatology, rheumatology, gastroenterology, and hematology. While effective, they can cause adverse effects such as acne, nausea, cytopenia, dyslipidemia, and Herpes zoster. Recent reports have linked JAKi usage to weight changes, particularly weight gain, which can significantly impact patients' quality of life. This study aimed to describe the incidence and characteristics of weight changes associated with the use of JAKi. Ovid MEDLINE, Embase, Web of Science, and Clinicaltrials.gov were searched up to April 2024. From 1080 initial articles, 90 studies covering 16,000 patients were selected. Our analysis found a notable incidence of weight gain with JAKi usage. Overall, 5.9% (947/16,000) of patients reported weight again. In randomized control trials, weight gain was observed in 7% (95% CI: 0.04; 0.09) of patients, while weight loss was observed in 1% (95% CI: 0.00; 0.03). Patients with dermatologic indications had lower weight gain rates (4%, 95% CI: 0.01; 0.06) than those with nondermatological indications (7%, 95% CI: 0.04; 0.10). Overall, JAKi therapy is associated with weight changes, particularly weight gain, underscoring the importance of appropriate counseling and weight monitoring. Further long-term studies are needed to better understand the mechanisms and management of JAKi-related weight changes.

## Introduction

Oral Janus kinase inhibitors (JAKi) and Tyrosine kinase-2 inhibitors have become increasingly popular for the treatment of various medical conditions. These include, but are not limited to alopecia areata, psoriasis, atopic dermatitis, vitiligo, rheumatoid arthritis, psoriatic arthritis, ankylosing spondylitis, inflammatory bowel disease, myelofibrosis, polycythemia vera, and graft-versus-host disease.^1^, ^2^, ^3^, ^4^ Several adverse effects have been documented with the use of JAKi such as acne, nausea, cytopenia, dyslipidemia, and Herpes zoster, among others.^5^ As the discontinuation of JAKi therapy is often prompted by adverse events rather than lack of efficacy, it is crucial to effectively manage these side effects.^6^

Interestingly, there are a growing number of reports linking the usage of oral JAKi with weight changes, particularly weight gain.^7^^,^^8^ One case series described rapid weight gain of up to 12.8 kg following JAKi initiation, leading to discontinuation of therapy.^9^ As unintended weight changes can significantly impact quality of life, a thorough understanding of this potential side effect of JAKi is crucial for physicians’ counselling and patients’ decision making.^10^ Despite the growing usage of JAKi in dermatology and other specialties for various indications, no comprehensive review of secondary weight changes has been conducted to date. This systematic review and meta-analysis aims to describe the incidence and characteristics of weight changes secondary to the use of various JAKi for all conditions, including dermatologic indications.

## Methods

This review follows the EQUATOR reporting guidelines (PRISMA 2020 checklist) for systematic reviews and was registered on PROSPERO: CRD42024548517.

### Search strategy

Ovid MEDLINE, Embase, Web of Science, Clinicaltrials.gov and preprint databases were searched on April 9, 2024 using variations of keywords (“Janus kinase inhibitors” or “JAKi” or “JAK inhibitor”) and (“weight changes” or “weight gain” or “weight loss.”) No date restrictions were used. The full search strategy can be found in (Supplementary Fig 1, available via Mendeley at https://data.mendeley.com/datasets/c7c84cxx3h/1).

### Eligibility

Inclusion criteria included (i) weight change as an outcome or adverse event of patients receiving JAKi for any indication, and (ii) observational, experimental studies, or case reports, with human subjects. Articles were excluded if JAKi therapy was given in combination with other non-JAKi medications known to cause weight changes (antipsychotics, corticosteroids, etc).

### Data screening

Articles were independently screened on COVIDENCE for titles, abstracts and full texts by 2 reviewers (E.Y and J.Y). Inter-rater reliability was strong with a rounded Cohen’s kappa of 0.8.^11^ Conflicts were resolved by discussion with a third reviewer (G.X.). Reference lists of included articles were checked to identify any additional studies. The study flowchart can be found in (Supplementary Fig 2, available via Mendeley at https://data.mendeley.com/datasets/c7c84cxx3h/1).

### Data extraction

Data extraction was completed by 4 authors (E.Y., J.Y, M.L, and M.H) after a pilot to ensure consistency. Extracted data were verified by another author (G. X.) and conflicts were resolved by consensus following a discussion. Study and patient characteristics, JAKi information, and weight change outcomes were extracted.

### Analysis

A random-effects model was used to calculate pooled effect sizes for weight gain and loss secondary to JAKi use. Meta-analysis was stratified by study type to minimize bias due to differences in study methodology. Subgroup analysis of three JAKi: ruxolitinib, upadacitinib, and tofacitinib, and dermatological versus nondermatological indications was conducted. Heterogeneity was assessed using the I^2^ statistic. All plots were generated on RStudio (Posit, PBC).

### Level of evidence

The Oxford Center for Evidence-Based Medicine 2011 Level of Evidence Table was used to determine the level of evidence in included articles.^12^ For observational studies, “dramatic effect” was defined as having a proportion of patients with weight changes larger than the mean of all included studies. Two authors (E.Y and M.L) completed the level of evidence appraisal, and disagreements were resolved through discussion with a third author (G.X).

## Results

### Study and patient characteristics

This study represents data from 16,000 patients (4774 male, 10,486 female, and 740 of unreported sex) across 90 articles. The weighted mean age of patients was 54.8 years (range: 16-92). Studies included 34 randomized control trials (RCTs), 24 observational, 12 nonrandomized, and 20 case reports. Weight change outcomes of patients recorded across the following JAKi: tofacitinib (*n* = 11,288), upadacitinib (*n* = 2230), ruxolitinib (*n* = 1887), filgotinib (*n* = 202), momelotinib (*n* = 104), fedratinib (*n* = 96), ilginatinib (48), abrocitinib (*n* = 41), lestaurtinib (*n* = 37), novel JAK-1 inhibitor INCB052793 (*n* = 36) and baricitinib (*n* = 29). Level of evidence analysis yielded 6 studies with level 1, 36 studies with level 2, 23 studies with level 3, and 25 studies which did not meet the Center for Evidence-Based Medicine criteria for assessment.

### Weight gain

Across all included studies, weight gain was reported in 5.9% (947/16,000) of patients. Weight gain events were reported 73.9 weeks after initiating JAKi therapy, on average. Among the 90 studies examined, 29 reported the magnitude of weight gain. The mean weight increase was 8.6 kg from a baseline mean weight of 63.4 kg, recorded at an average of 74.3 weeks into treatment.

**Fig 1:**
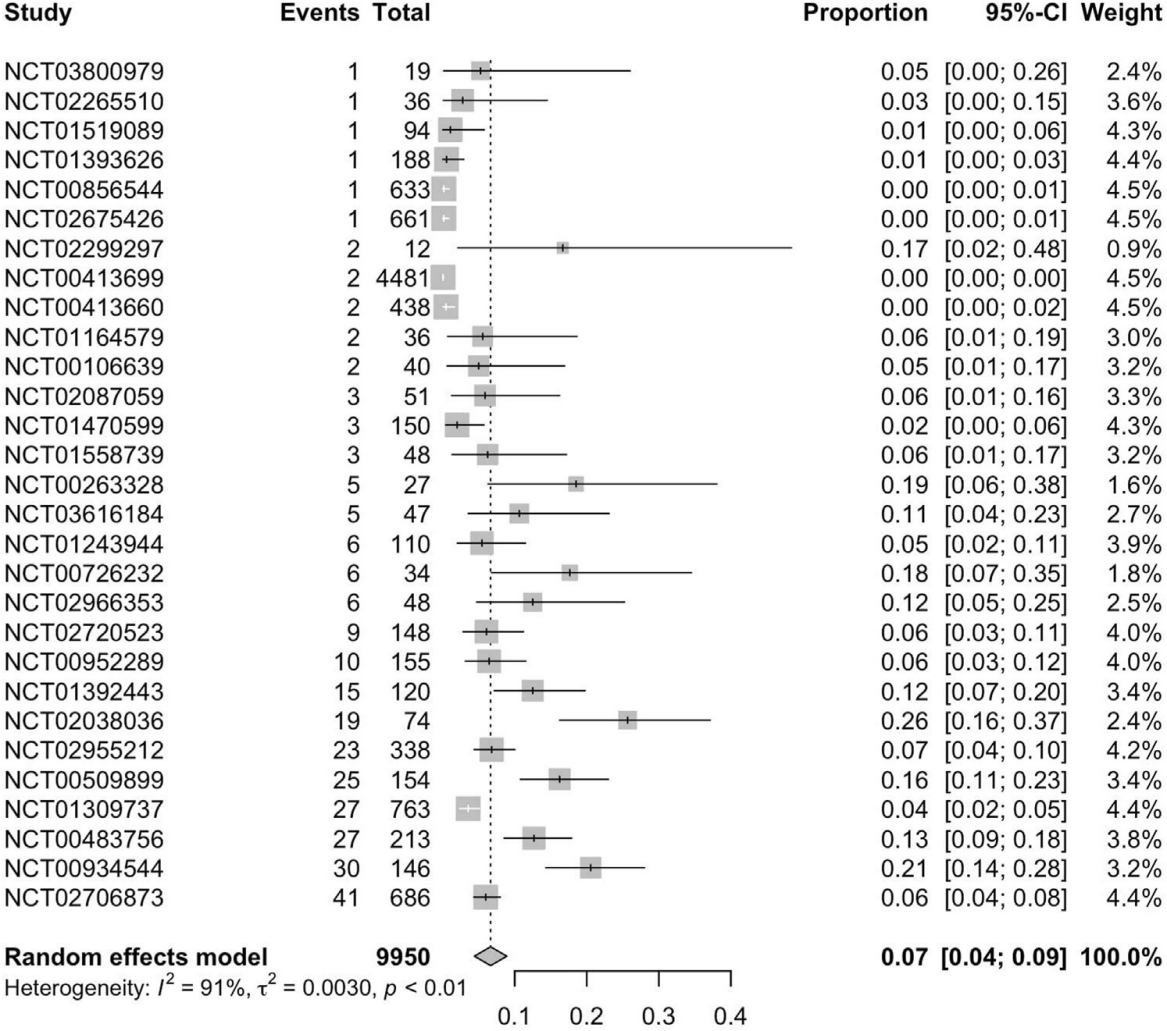
Pooled effect size of weight gain secondary to oral Janus kinase inhibitor use in randomized control trials.

**Fig 2:**
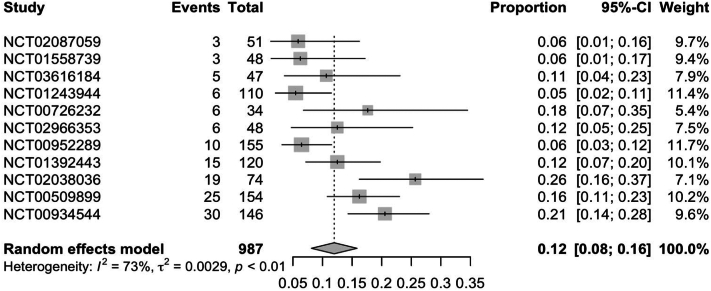
Pooled effect size of weight gain secondary to oral ruxolitinib in randomized control trials.

**Fig 3:**
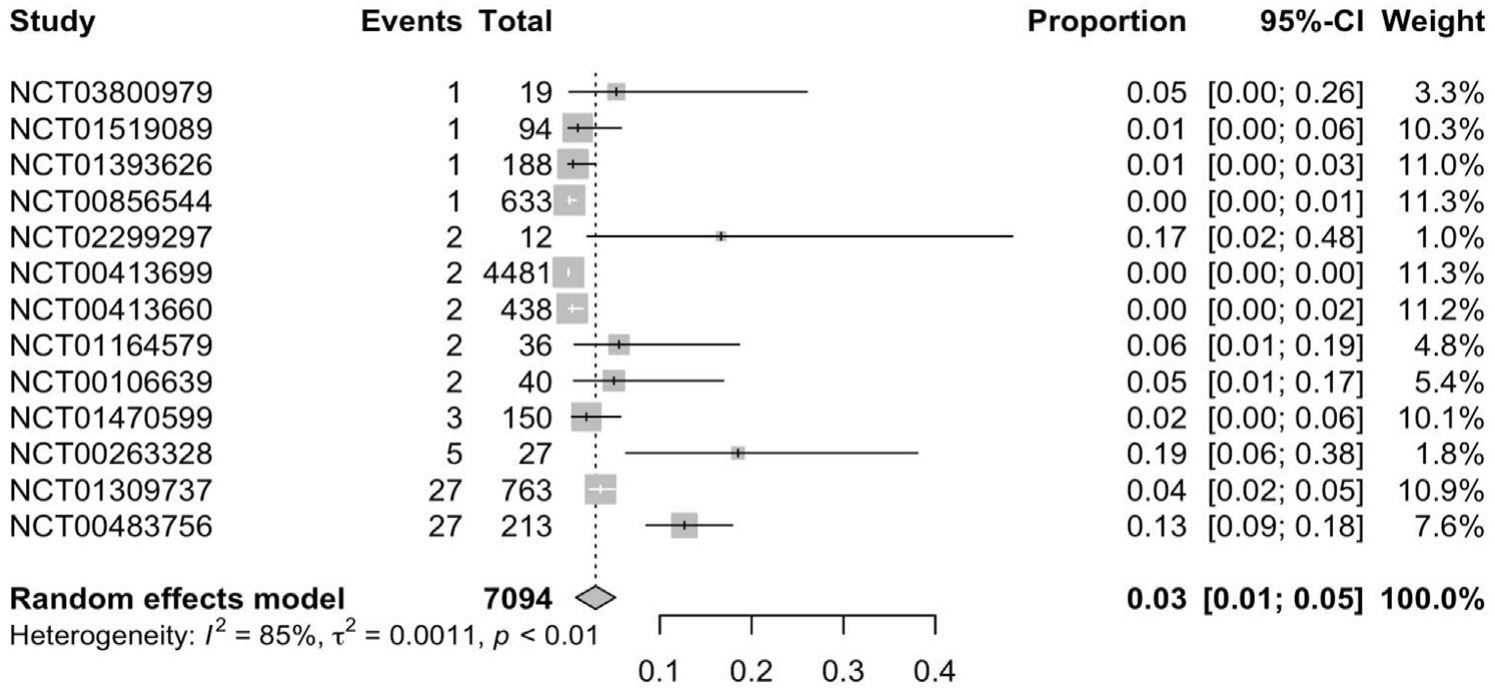
Pooled effect size of weight gain secondary to tofacitinib in randomized control trials.

**Fig 4:**
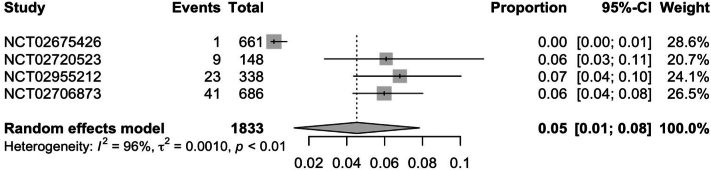
Pooled effect size of weight gain secondary to oral upadacitinib in randomized control trials.

**Fig 5:**
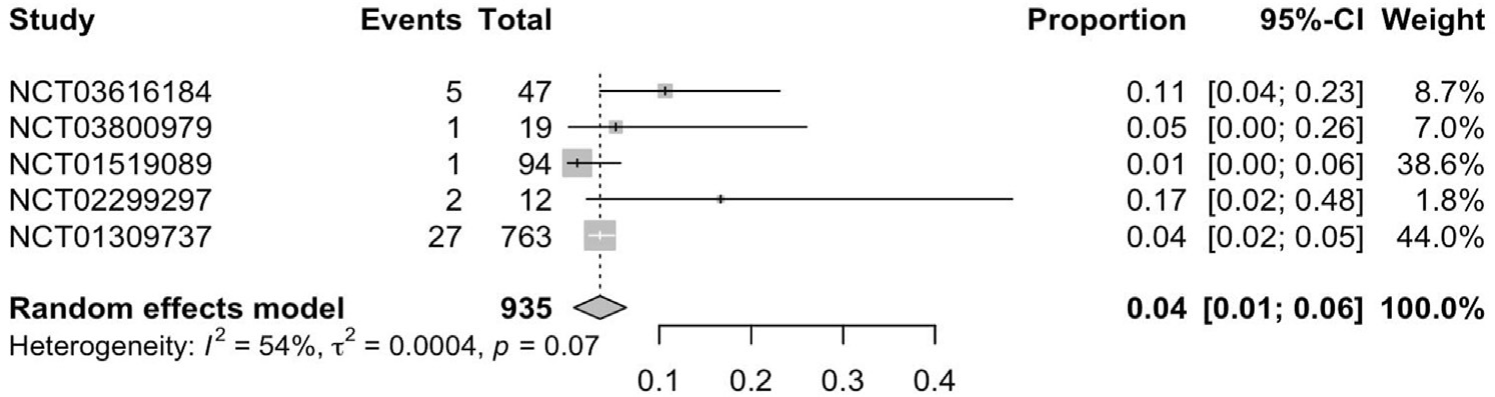
Pooled effect size of weight gain secondary to oral Janus kinase inhibitor use in dermatologic indications in randomized control trials.

**Fig 6:**
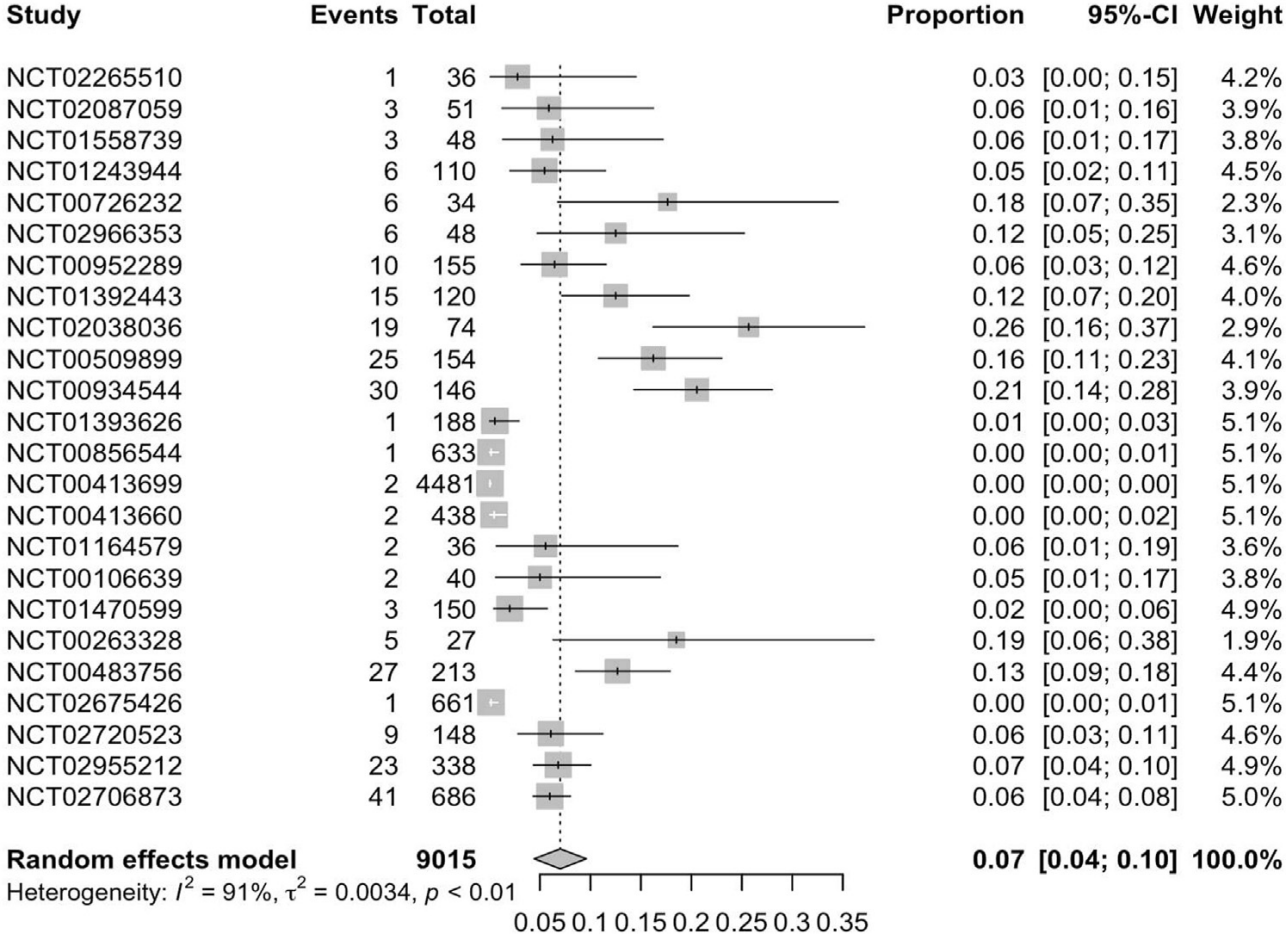
Pooled effect size of weight gain secondary to Janus kinase inhibitor use in nondermatologic indications in randomized control trials.

In RCTs, weight gain secondary to JAKi across all indications was observed in 7.0% (95% CI: 0.04; 0.09) of patients (Fig 1). Further subgroup analysis of RCTs found ruxolitinib to have the highest rate of weight gain at 12% (95% CI: 0.08; 0.16), followed by upadacitinib [5% (95% CI: 0.01; 0.08)] and tofacitinib [3% (95% CI: 0.01; 0.05)] (Fig 2, Fig 3, Fig 4). Notably, dermatologic indications had lower rates of weight gain than nondermatological indications, at 4.0% (95% CI: 0.01; 0.06) compared to 7% (95% CI: 0.04; 0.10) of patients (Figs 5 and 6). Numerous trials (NCT01243944, NCT00952289, and NCT02038036) also demonstrated higher rates of weight gain in patients treated with JAKi compared to placebos and controls of the best available therapy. Notably, NCT01309737, a large-scale trial for tofacitinib in plaque psoriasis, found zero cases of weight gain in the placebo group compared to 3.54% (27/763) of the intervention group.^13^

**Fig 7:**
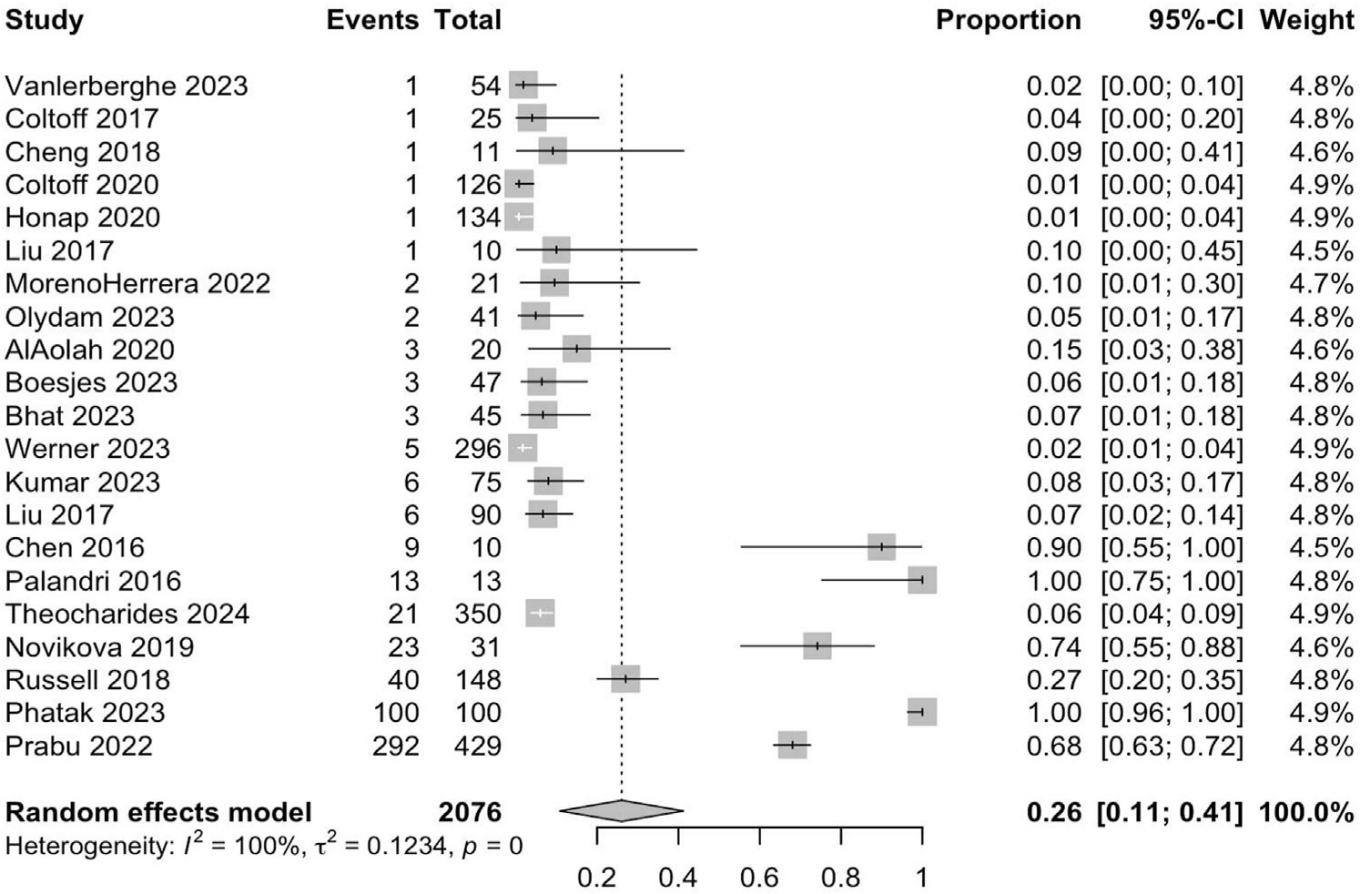
Pooled effect size of weight gain secondary to Janus kinase inhibitor use in observational studies.

**Fig 8:**
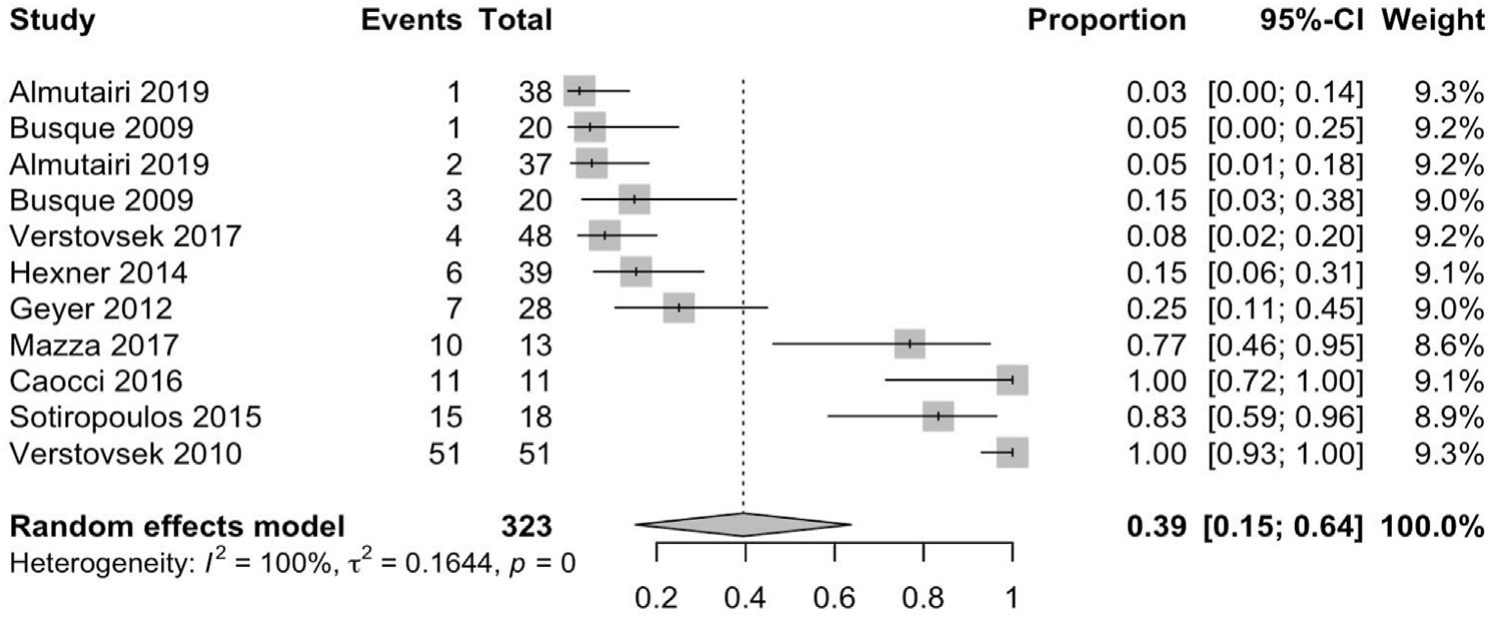
Pooled effect size of weight gain secondary to Janus kinase inhibitor use in nonrandomized studies.

Pooled results for observational and nonrandomized studies showed larger rates of weight gain, at 26% (95% CI: 0.11; 0.41) and 39% (95% CI: 0.15; 0.64), respectively (Figs 7 and 8). However, these effect sizes may be biased due to poor control of confounding variables and discrepancies between study methodologies, compared to RCTs.

### Weight loss

**Fig 9:**
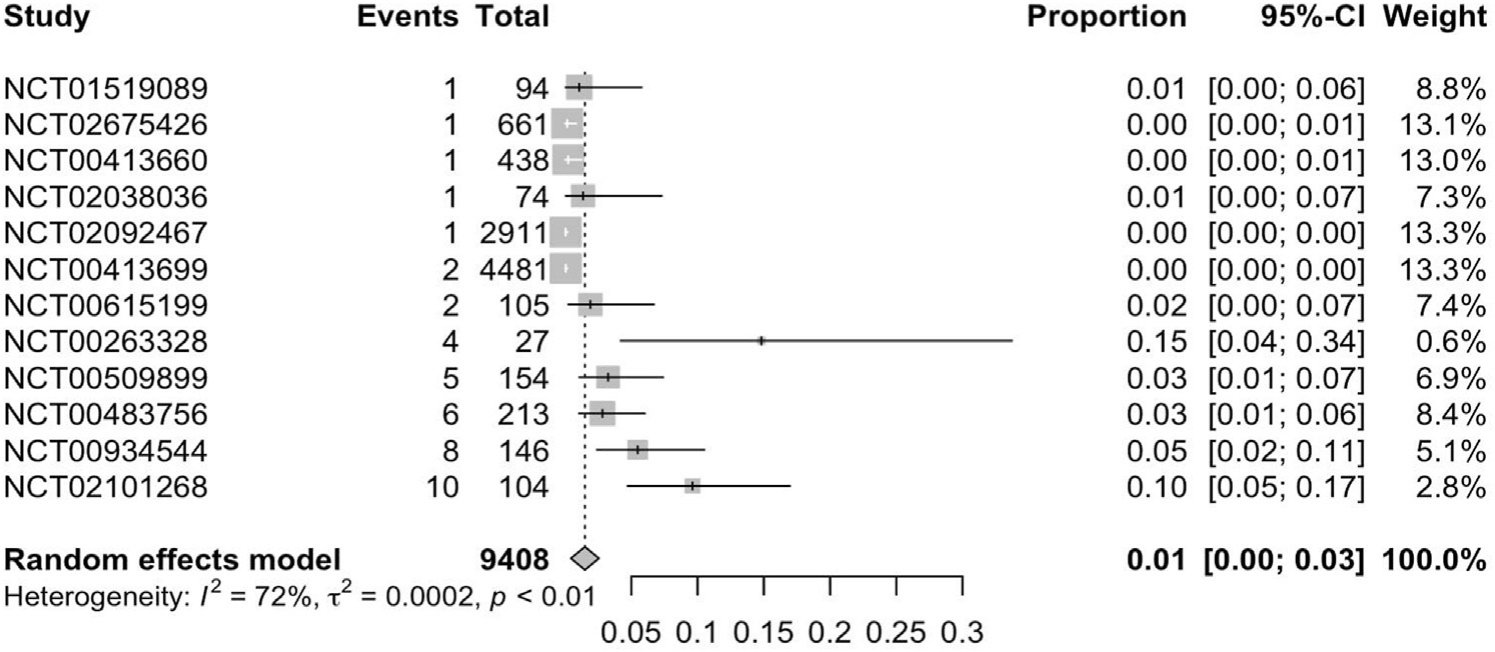
Pooled effect size of weight loss secondary to Janus kinase inhibitor use in randomized control trials.

On the other hand, weight loss was reported in 1.0% (158/16,000) of patients across all included studies. Only one case report quantified weight loss secondary to JAKi use, at 1.5 kg from 110.6 kg. In RCTs, weight loss secondary to JAKi usage across all indications was seen in 1.0% (95% CI: 0.00; 0.03) of patients (Fig 9). Weight loss in other study types was not substantial enough for meta-analysis. Notably, no articles reported weight loss secondary to JAKi use in dermatologic indications.

## Discussion

This systematic review and meta-analysis comprehensively evaluated weight changes associated with JAKi usage in the literature. Overall, weight gain was observed in 5.9% of patients across all study types and indications. Particularly, patients with dermatologic indications exhibited lower rates of weight gain (4.0%) compared to those with nondermatological indications (7.0%) when using JAKi.

While the exact mechanism behind JAKi-associated weight gain remains unclear, several theories have been proposed. Some evidence suggests that JAKi may increase skeletal muscle mass, leading to subsequent weight gain. One study found that rabbits treated with tofacitinib experienced increased muscle mass and weight gain, which the authors attributed to muscle remodeling.^14^ Asymptomatic elevation of serum creatine kinase and creatinine levels has been observed in multiple JAKi clinical trials for various inflammatory diseases, further indicating a potential role in muscle remodeling.^15^^,^^16^ Moreover, muscle catabolism is common in patients with systemic inflammatory diseases. This is linked to certain inflammatory cytokines that cause muscle wasting and increased resting energy expenditure, a phenomenon well documented in calorimetry studies.^17^ Accordingly, numerous studies using animal and in-vitro engineered human muscle models have demonstrated that inhibition of the JAK/STAT pathway can reduce IL-6 and TNF-α induced muscle wasting.^18^^,^^19^ Given that muscle and metabolic issues are more prominent in nondermatological inflammatory conditions such as rheumatoid arthritis and myelofibrosis, patients may experience more significant weight gain when the JAK/STAT pathway is inhibited due to reduced muscle wasting. This contrasts with dermatological conditions, where the JAK/STAT pathway’s role in muscle metabolism is likely less significant. This difference may explain the higher rates of weight gain secondary to JAKi use observed in patients with nondermatological conditions compared to dermatological conditions in our study. Additionally, Price et al found that hyperactive JAK/STAT signaling due to age or disease state impairs myogenic proliferation, differentiation, and self-renewal.^20^ Their research demonstrates the potential of JAKi-use to restore muscle regeneration, a mechanism which can be leveraged therapeutically. For instance, JAKi-use has previously been linked to improved cachexia amongst patients with myeloproliferative disorders.^21^

Although weight gain was the more common outcome secondary to JAKi use, weight loss was also observed in some patients. This seemingly paradoxical weight loss could be explained by the gastrointestinal side effects of oral JAKi, particularly nausea. Nausea and other GI side effects have been reported with nearly all JAKi, though at variable rates.^22^ GI side effects may affect appetite and lead to reduced food intake, thereby inciting the observed weight loss.^21^ As proinflammatory cytokines have been linked to obesity-related insulin resistance and the activation of adipokines, another possible theory behind JAKi-associated weight loss is that reduced levels of IL-6 via JAK inhibition might lead to decreased adiposity.^23^^,^^24^ An in-vivo study conducted by Qurania et al supports this hypothesis, where JAKi-administered mice on a high-fat diets maintained similar levels of adiposity as controls. This effect was attributed to the induction of white adipose tissue browning, further underscoring the potential of JAK inhibition in preserving metabolic health.^25^

Moreover, the successful treatment of inflammatory arthropathies with JAKi often leads to reduced pain, stiffness, and overall enhanced ambulation in patients with rheumatic diseases.^26^ These patients may also experience significant improvement to their mood and quality of life, factors often associated with increased social and outdoor activity.^26^ Accordingly, increased mobility and physical activity levels may explain the observed weight loss after JAKi use.^24^

While weight loss has also been reported in nonhematologic conditions, unintended weight loss is common in patients with hematologic disorders. This typically occurs post stem-cell and bone marrow transplantation, and in patients with graft-versus-host disease, regardless of JAKi usage.^27^^,^^28^ This may have contributed to the observed weight loss in patients receiving oral ruxolitinib, a JAKi commonly used to treat hematologic disease, and to the overall weight loss associated with JAKi therapy in this analysis. Furthermore, this tendency could have also obscured cases of true weight gain, particularly as weight outcomes were self-reported in most studies. Additionally, the duration of JAKi treatment and timing of when weight changes were recorded were not consistently reported across all included studies. These discrepancies could have led to an overrepresentation of weight loss in our results, as there may not have been sufficient time for JAKi-related increases in muscle mass and weight to manifest.

Although secondary weight changes might be undesirable for some patients, we argue that they also highlight a beneficial aspect of JAK inhibitors. Specifically, our findings emphasize the therapeutic potential of JAKi in treating metabolic syndrome by increasing muscle mass and reducing fat. This is achieved by addressing the inflammatory states associated with chronic diseases and aging.

Ultimately, the immense molecular crosstalk within inflammatory pathways and diversity in indications and patients receiving JAKi therapy creates substantial challenges in understanding the mechanisms behind JAKi-associated weight changes. Nonetheless, increased education about weight changes secondary to JAKi use in patient-specific contexts will improve patients’ ability to make informed decisions about initiating treatment.^29^ Awareness of JAKi-associated weight changes may also impact patient counselling regarding necessary lifestyle modifications such as exercise, sleep, and protein intake.

To our knowledge, this is the first large-scale systematic review and meta-analysis investigating weight changes secondary to JAKi use. Strengths of this review include its novelty and incorporation of data from various levels of evidence, ranging from RCTs to case reports. This has allowed for a comprehensive review of all instances of weight changes secondary to JAKi in the literature to date. However, the interpretation of our results must also be considered within the limitations of our review. Firstly, significant heterogeneity was present in the included studies, and our results may have been affected by publication bias due to the underreporting of smaller effect sizes (Supplementary Fig 3, available via Mendeley at https://data.mendeley.com/datasets/c7c84cxx3h/1). Secondly, weight changes were often reported as secondary outcomes in the included articles, leaving potential for reporting and other biases. Thirdly, we were unable to investigate differences between male and female populations due to the absence of stratified data in the included studies. While our review attempted to only include cases of JAKi monotherapy, weight changes may have also been impacted by unreported comorbidities or other weight-altering drugs and behaviors. Lastly, most studies did not specify the time at which weight changes were observed, or the magnitude of any changes, further limiting our understanding of the short-versus long-term effects of JAKi use.

Future research should focus on better characterizing weight changes associated with JAKi use, particularly in male versus female populations, and in pediatric patients. Studies observing JAK/STAT-driven differences in signaling molecule activity and effects on skeletal muscle and adipose tissue are also of particular interest. Clinical studies should also prioritize the collection of weight change data, preferably as a study outcome instead of adverse event to combat publication and self-reporting bias. Such investigations would ideally be participant-blinded to limit any weight-altering behaviors that may affect results,^30^ and rely on measurable, as opposed to patient-reported assessments to reduce reporting bias.

## Conclusion

Use of JAKi is associated with weight changes, particularly weight gain. Awareness of this association is crucial for patient counselling and may lead to expanded therapeutic use of JAKi metabolic disease. Long-term studies with standardized weight monitoring protocols are needed to better understand the mechanisms and management of JAKi-related weight changes.

## Learning points

• This study was undertaken to characterize the incidence and nature of weight changes secondary to JAKi use for various indications
• Our results demonstrated that JAKi is associated with weight changes, specifically weight gain in 5.9% and weight loss in 1.0% of patients. Weight gain secondary to JAKi use was higher in patients with nondermatological indications, compared to dermatologic indications.
• Increased awareness of JAKi-associated weight gain may be significant for patient counselling and decision making
• This study has implications for future exploration of the therapeutic potential of JAKi in cachexia and metabolic disease

## Conflicts of interest

Dr Abu-Hilal has been speaker, advisor and/or received honoraria from AbbVie, Biojamp, Boehringer Ingelheim, Bristol Myers Squibb, Celltrion, Eli Lilly, Galderma, Hikma Pharmaceuticals, Incyte, Janssen, Leo, L’Oreal, La Roche Posay, Medexus, Novartis, Pfizer, Recordati, Sanofi Regeneron, and Sun Pharma. Authors Xiong, Yu, Heung, Yang, and Lowe have no conflicts of interest to declare.

## References

[1] Roskoski R. (2023). Deucravacitinib is an allosteric TYK2 protein kinase inhibitor FDA-approved for the treatment of psoriasis. Pharmacol Res.

[2] Cunningham K.N., Rosmarin D. (2023). Vitiligo treatments: review of current therapeutic modalities and JAK inhibitors. Am J Clin Dermatol.

[3] Gupta A.K., Wang T., Polla Ravi S., Bamimore M.A., Piguet V., Tosti A. (2023). Systematic review of newer agents for the management of alopecia areata in adults: janus kinase inhibitors, biologics and phosphodiesterase-4 inhibitors. J Eur Acad Dermatol Venereol.

[4] Dogra S., Sharma A., Mehta H., Sarkar R. (2023). Emerging role of topical Janus kinase inhibitors in dermatological disorders: a review. Clin Exp Dermatol.

[5] Hoisnard L., Lebrun-Vignes B., Maury S. (2022). Adverse events associated with JAK inhibitors in 126,815 reports from the WHO pharmacovigilance database. Sci Rep.

[6] Aymon R., Mongin D., Bergstra S.A. (2024). Evaluation of discontinuation for adverse events of JAK inhibitors and bDMARDs in an international collaboration of rheumatoid arthritis registers (the ‘JAK-pot’ study). Ann Rheum Dis.

[7] Ch’en P.Y., Ng J., Song E.J. (2023). Weight gain secondary to the use of Janus kinase inhibitors. Arch Dermatol Res.

[8] Sapre M., Tremblay D., Wilck E. (2019). Metabolic effects of JAK1/2 inhibition in patients with myeloproliferative neoplasms. Sci Rep.

[9] Shah K., Shukla D., Patel M., Malhotra S. (2023). A case series on tofacitinib-induced weight gain. Indian J Pharmacol.

[10] Stafford M., Hemingway H., Marmot M. (1998). Current obesity, steady weight change and weight fluctuation as predictors of physical functioning in middle aged office workers: the Whitehall II Study. Int J Obes Relat Metab Disord.

[11] McHugh M.L. (2012). Interrater reliability: the kappa statistic. Biochem Med.

[12] OCEBM Levels of Evidence Working Group∗ “The Oxford Levels of Evidence 2”. Oxford Centre for Evidence-Based Medicine. https://www.cebm.ox.ac.uk/resources/levels-of-evidence/ocebm-levels-of-evidence.

[13] Papp K.A., Menter M.A., Abe M., OPT Pivotal 2 Investigators (2015). Tofacitinib, an oral Janus kinase inhibitor, for the treatment of chronic plaque psoriasis: results from two randomized, placebo-controlled, phase III trials. Br J Dermatol.

[14] Bermejo I., Pérez-Baos S., Medina J.P. (2022). Ab0064 pharmacological inhibition of Il-6/Jak/Stat axis increases muscle mass in an experimental model of sarcopenia associated to rheumatoid arthritis. Ann Rheum Dis.

[15] Panaccione R., Isaacs J.D., Chen L.A. (2021). Characterization of creatine kinase levels in tofacitinib-treated patients with ulcerative colitis: results from clinical trials. Dig Dis Sci.

[16] Isaacs J.D., Zuckerman A., Krishnaswami S. (2014). Changes in serum creatinine in patients with active rheumatoid arthritis treated with tofacitinib: results from clinical trials. Arthritis Res Ther.

[17] Hanaoka B.Y., Zhao J., Heitman K. (2022). Interaction effect of systemic inflammation and modifiable rheumatoid cachexia risk factors on resting energy expenditure in patients with rheumatoid arthritis. JCSM Clin Rep.

[18] Chen Z., Li B., Zhan R.Z., Rao L., Bursac N. (2021). Exercise mimetics and JAK inhibition attenuate IFN-γ–induced wasting in engineered human skeletal muscle. Sci Adv.

[19] Bonetto A., Aydogdu T., Jin X. (2012). JAK/STAT3 pathway inhibition blocks skeletal muscle wasting downstream of IL-6 and in experimental cancer cachexia. Am J Physiol Endocrinol Metab.

[20] Price F.D., von Maltzahn J., Bentzinger C.F. (2014). Inhibition of JAK-STAT signaling stimulates adult satellite cell function. Nat Med.

[21] Breccia M., Bartoletti D., Bonifacio M. (2019). Impact of comorbidities and body mass index in patients with myelofibrosis treated with ruxolitinib. Ann Hematol.

[22] Song Y.K., Song J., Kim K., Kwon J.W. (2022). Potential adverse events reported with the Janus kinase inhibitors approved for the treatment of rheumatoid arthritis using spontaneous reports and online patient reviews. Front Pharmacol.

[23] Ouchi N., Parker J.L., Lugus J.J., Walsh K. (2011). Adipokines in inflammation and metabolic disease. Nat Rev Immunol.

[24] Kirichenko T.V., Markina Y.V., Bogatyreva A.I., Tolstik T.V., Varaeva Y.R., Starodubova A.V. (2022). The role of adipokines in inflammatory mechanisms of obesity. Int J Mol Sci.

[25] Qurania K.R., Ikeda K., Wardhana D.A. (2018). Systemic inhibition of Janus kinase induces browning of white adipose tissue and ameliorates obesity-related metabolic disorders. Biochem Biophys Res Commun.

[26] de Souza S., Williams R., Nikiphorou E. (2024). Clinician and patient views on Janus kinase inhibitors in the treatment of inflammatory arthritis: a mixed methods study. BMC Rheumatology.

[27] Szeja N., Grosicki S. (2020). Refeeding syndrome in hematological cancer patients – current approach. Expert Rev Hematol.

[28] Jacobsohn D.A., Margolis J., Doherty J., Anders V., Vogelsang G.B. (2002). Weight loss and malnutrition in patients with chronic graft-versus-host disease. Bone Marrow Transplant.

[29] Nash P., Kerschbaumer A., Dörner T. (2021). Points to consider for the treatment of immune-mediated inflammatory diseases with Janus kinase inhibitors: a consensus statement. Ann Rheum Dis.

[30] Ji Y., Huang Q., Liu H., Phillips C. (2021). Weight bias 2.0: the effect of perceived weight change on performance evaluation and the moderating role of anti-fat bias. Front Psychol.

